# Comparison of next‐generation portable pollution monitors to measure exposure to PM_2.5_ from household air pollution in Puno, Peru

**DOI:** 10.1111/ina.12638

**Published:** 2020-01-23

**Authors:** Vanessa J. Burrowes, Ricardo Piedrahita, Ajay Pillarisetti, Lindsay J. Underhill, Magdalena Fandiño‐Del‐Rio, Michael Johnson, Josiah L. Kephart, Stella M. Hartinger, Kyle Steenland, Luke Naeher, Katie Kearns, Jennifer L. Peel, Maggie L. Clark, William Checkley, HAPIN Investigators, Abidan Nambajimana, Amit Verma, Amy Lovvorn, Anaité Diaz, Aris Papageorghiou, Ashley Toenjes, Ashlinn Quinn, Azhar Nizam, Barry Ryan, Bonnie Young, Dana Barr, Dina Goodman, Eduardo Canuz, Elisa Puzzolo, Eric McCollum, Erick Mollinedo, Fiona Majorin, Florien Ndagijimana, Ghislaine Rosa, Gurusamy Thangavel, Howard Chang, Irma Sayury Pineda Fuentes, J. Jaime Miranda, Jean de Dieu Ntivuguruzwa, Jean Uwizeyimana, Jennifer Peel, Jeremy Sarnat, Jiawen Liao, John McCracken, Joshua Rosenthal, Juan Gabriel Espinoza, Julia McPeek Campbell, Kalpana Balakrishnan, Kendra Williams, Kirk Smith, Krishnendu Mukhopadhyay, Lance Waller, Lawrence Moulton, Lindsay Jaacks, Lisa Elon, Lisa Thompson, Margaret Laws, Marilú Chiang, Marjorie Howard, Mary Crocker, Miles Kirby, Naveen Puttaswamy, Oscar De Leon, Phabiola Herrera, Rachel Craik, Rachel Merrick, Sankar Sambandam, Sarada Garg, Sarah Rajkumar, Savannah Gupton, Shakir Hossen, Sheela Sinharoy, Shirin Jabbarzadeh, Steven Harvey, Suzanne Simkovich, Thomas Clasen, Usha Ramakrishnan, Victor G Davila‐Roman, Vigneswari Aravindalochanan, Yunyun Chen, Zoe Sakas

**Affiliations:** ^1^ Division of Pulmonary and Critical Care Johns Hopkins University School of Medicine Baltimore MD USA; ^2^ Center for Global Non‐Communicable Disease Research and Training School of Medicine Johns Hopkins University Baltimore MD USA; ^3^ Department of International Health Johns Hopkins Bloomberg School of Public Health Baltimore MD USA; ^4^ Berkeley Air Monitoring Group Berkeley CA USA; ^5^ Environmental Health Sciences University of California Berkeley Berkeley CA USA; ^6^ Department of Environmental Health Emory University Rollins School of Public Health Atlanta GA USA; ^7^ Department of Environmental Health and Engineering Johns Hopkins Bloomberg School of Public Health Baltimore MD USA; ^8^ Facultad de Salud Pública y Administración Universidad Peruana Cayetano Heredia Lima Peru; ^9^ Swiss Tropical and Public Health Institute Basel Switzerland; ^10^ Department of Environmental Health Sciences University of Georgia College of Public Health Athens GA USA; ^11^ Department of Environmental and Radiological Health Sciences Colorado State University CO USA

**Keywords:** exposure assessment, fine particulate matter, household air pollution, instrument validation, lower‐ and middle‐income countries, personal exposure

## Abstract

Assessment of personal exposure to PM_2.5_ is critical for understanding intervention effectiveness and exposure‐response relationships in household air pollution studies. In this pilot study, we compared PM_2.5_ concentrations obtained from two next‐generation personal exposure monitors (the Enhanced Children MicroPEM or ECM; and the Ultrasonic Personal Air Sampler or UPAS) to those obtained with a traditional Triplex Cyclone and SKC Air Pump (a gravimetric cyclone/pump sampler). We co‐located cyclone/pumps with an ECM and UPAS to obtain 24‐hour kitchen concentrations and personal exposure measurements. We measured Spearmen correlations and evaluated agreement using the Bland‐Altman method. We obtained 215 filters from 72 ECM and 71 UPAS co‐locations. Overall, the ECM and the UPAS had similar correlation (ECM ρ = 0.91 vs UPAS ρ = 0.88) and agreement (ECM mean difference of 121.7 µg/m^3^ vs UPAS mean difference of 93.9 µg/m^3^) with overlapping confidence intervals when compared against the cyclone/pump. When adjusted for the limit of detection, agreement between the devices and the cyclone/pump was also similar for all samples (ECM mean difference of 68.8 µg/m^3^ vs UPAS mean difference of 65.4 µg/m^3^) and personal exposure samples (ECM mean difference of −3.8 µg/m^3^ vs UPAS mean difference of −12.9 µg/m^3^). Both the ECM and UPAS produced comparable measurements when compared against a cyclone/pump setup.

**Practical Implications**

- We conducted this study in Puno, Peru, to evaluate the ability of two exposure assessment instruments to collect particulate matter concentrations from household air pollution and compare them against a traditional cyclone and pump method.
- These relatively less expensive, lighter, and easier‐to‐use instruments, as compared to traditional instruments, may enable higher spatiotemporal resolution for air quality monitoring in real‐world field settings.
- Thus, this will help improve exposure‐response estimates for health outcomes and exposure classification, and inform health program planning, future intervention evaluation, and chronic disease management.

## 1 INTRODUCTION

Household air pollution (HAP) adversely affects nearly three billion people who use open fires and biomass fuels as their main sources of fuel for cooking.^1^, ^2^, ^3^ Burning biomass fuels, particularly in poorly ventilated cooking areas, can emit dangerously high concentrations of pollutants such as particulate matter, black carbon, and carbon monoxide into the household environment.^2^ A critical cut‐point size of particulate matter is less than 2.5 micrometers in aerodynamic diameter (PM_2.5_), due to its ability to penetrate lung tissue, traverse tissue barriers, and ultimately enter the bloodstream to cause systemic inflammation.^4^ The World Health Organization (WHO) estimates that four million premature deaths and 45% of pneumonia deaths in children less than five years of age can be attributed to HAP exposure from biomass fuel use.^5^ Pregnant women and their young children are especially vulnerable due to the large amount of time spent indoors cooking.^6^ These include higher risk of low birthweight, increased stillbirth,^7^, ^8^ higher odds of acute lower respiratory infections among children less than five years of age,^9^ and higher risk of perinatal mortality, neonatal mortality, and macerated stillbirths.^8^, ^10^

Few studies, however, have adequately measured personal exposure to household air pollution because of lack of easy‐to‐use and easy‐to‐deploy equipment.^11^, ^12^ In past studies, intention‐to‐treat analyses lacking personal exposure measurements have led to exposure misclassification and underestimating the true effect of HAP exposure on health outcomes due to stove stacking.^13^ Attempts to formulate exposure‐response curves for HAP and personal exposure to PM_2.5_ against respiratory health outcomes have been limited by the lack of data. Smith and Peel^14^ drew attention to the lack of HAP personal exposure data used in exposure‐response analyses. For example, the integrated exposure‐response analyses developed by Burnett et al^15^ uses only a limited amount of HAP data. As of today, the situation has not greatly improved. Overall, systematic measurement of personal exposure to PM_2.5_ in conjunction with specific health outcomes is strongly needed to increase precision of exposure‐response estimates and better evaluate the impacts of cookstove and clean fuel interventions in the future.

There continues to be poor assessment of personal exposure in previous cookstove studies. For example, the Randomized Exposure Study of Indoors and Respiratory Effects Study (RESPIRE) in Guatemala did not measure personal PM_2.5_ exposure measurements and used carbon monoxide (CO) exposure instead to approximate particulate matter exposure rather than direct PM_2.5_ exposure assessment. While the relationship between CO and PM_2.5_ was well‐validated for the specific setting of the Western Highlands in Guatemala, this relationship has been shown to have poor‐to‐moderate correlation in other contexts.^16^, ^17^

Traditional pump and cyclone approaches for assessing personal exposure include a battery‐powered sampling pump typically worn on the hip or in a backpack with tubing attached to a particle size‐selective device installed in the human breathing zone.^11^, ^18^ These setups are generally expensive, noisy, heavy, and bulky for personal exposure measurements on women and children^19^, ^20^ and are often filter‐based, requiring laboratory analysis and delaying results for study participants and subsequent analyses. Therefore, there is a strong need to validate lighter, lower‐cost, and easier‐to‐use tools for air quality monitoring in low‐resource field settings.^21^, ^22^ These instruments will thus improve measurement of an individual's personal air pollution exposure and exposure classification in their home, exposure‐response estimates for health outcomes, and ultimately data resolution in low‐ to middle‐income countries for health program planning, future intervention evaluation, and chronic disease management.^23^

## 2 METHODS

### 2.1 Study setting

The Household Air Pollution Intervention Network (HAPIN) trial, the parent study to the work described here, is a randomized controlled trial of a liquefied petroleum gas (LPG) stove and continuous fuel distribution in 3200 households in four LMICs (India, Guatemala, Rwanda, and Peru) (https://clinicaltrials.gov/ct2/show/NCT02944682). This pilot study took place in Puno, Peru, which lies at an altitude of 3825 meters above sea level and has low temperatures (average range of 5.9°C‐9.8°C) throughout most of the year.^24^ Most communities in this region speak Spanish, Aymara, and Quechua languages, and use wood, dung, and crop residues as cooking and heating fuels in traditional stoves (known as fogón in Spanish).

### 2.2 Study design

**Figure 1:**
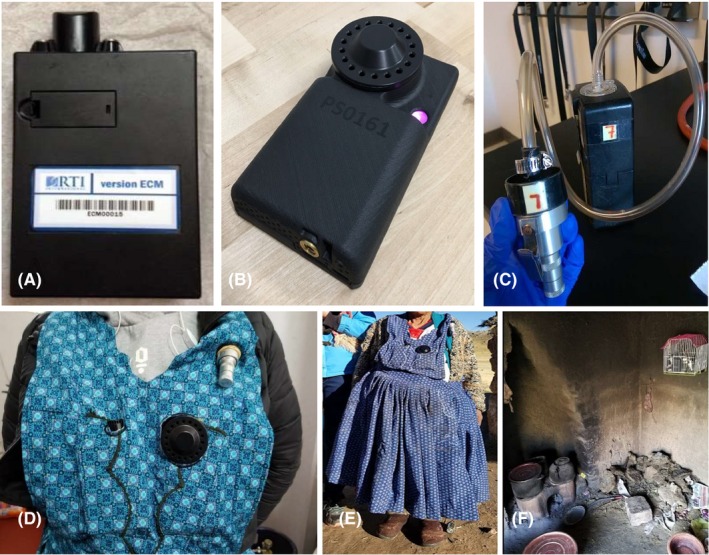
(From left to right) – A.) Enhanced Children's MicroPEM (ECM), B.) Ultrasonic Personal Air Sampler (UPAS [photograph from Access Sensor Technologies]), and C.) Triplex Personal Sampling Cyclone with SKC AirChek XR5000 pump [Mesa Labs and SKC]); D.) Shown left to right, the ECM, UPAS, and Cyclone air inlets are co‐located on a customized study apron with holes shaped to the instruments; E.) Medium‐exposure study participant wearing personalized apron with all three co‐located exposure instruments; F.) In‐house installation of birdcage containing ECM, UPAS, and Cyclone machines

We sought to test the wearability of two new devices and agreement with a cyclone/pump PM_2.5_ exposure assessment equipment. The Enhanced Children MicroPEM (ECM, RTI International) is a recently developed instrument that is a relatively lower‐cost, easier‐to‐use PM_2.5_ gravimetric, and real‐time exposure assessment tool when compared to a cyclone/pump setup (Figure 1A). The ECM is the size of a pack of cards, weighs about 150 grams (RTI International), has a flow rate of 0.3 L/min, short battery charging times (4‐6 hours), and uses small 15 mm filters. This instrument can also measure particle concentration via nephelometric particle scattering, participant compliance with an accelerometer, and it logs temperature, relative humidity, and flow rate. An accelerometer is useful in measuring participant compliance in wearing sampling instrumentation, as it can detect participant activity levels to confirm that the participant is physically wearing the device.^25^, ^26^ The second instrument evaluated in this study, the Ultrasonic Personal Air Sampler (UPAS; Access Sensor Technologies, Fort Collins, CO) (Figure 1B), weighs 230 grams, has a flow rate of 1 L/min, can be charged within three and a half to four hours, and is about the size of a standard smartphone (128 × 70 × 33 mm). The UPAS collects PM_2.5_ data gravimetrically on 37 mm filters and collects motion data via an accelerometer, temperature, and humidity data. It can also be programmed to start sampling within certain GPS coordinates and has an Android/iPhone application for instrument configuration, flow rate, programming of sample characteristics, and downloading of data (UPAS v2.1.9; Access Sensor Technologies). Both instruments were used to collect filter‐based PM_2.5_ samples to compare against gravimetric samples collected by a currently accepted traditional pump and cyclone setup. In this study, we used a Triplex Personal Sampling Cyclone (Mesa Labs) with a SKC AirChek XR5000 pump (SKC) with a weight of 450 grams and sampling at 1.5 L/min. This setup has been used frequently in previous validation studies^18^, ^25^, ^27^, ^28^ (Figure 1C).

### 2.3 Exposure assessment

Eighty‐two co‐location PM_2.5_ HAP measurements were collected with the three co‐located instruments (ECM, UPAS, and the cyclone/pump). To evaluate the range of PM concentrations typical of rural households using biomass or gas stoves, we used three different classifications of 24‐hour HAP exposures (low, medium, and high pollution environments, as described below) and collected PM_2.5_ gravimetrically with all three instruments.^29^

The primary motivation for this study was to compare the agreement of the ECM and UPAS with the cyclone/pump setup at low‐exposure concentrations that can contribute to the lower end of the exposure‐response curve, as little data currently exists for this range.^11^, ^14^, ^30^ Previous studies looking at different airborne particulate exposures and similar health endpoints have conflicting findings, leading to a recommendation to improve exposure classification to define specific exposure cut‐points on the response curves that may help design better interventions and formulate policy.^11^, ^14^, ^30^ Additionally, these lower exposures may be representative of 24‐hour exposures in homes using clean fuels. To the best of our knowledge, this will be the first time this range of concentrations has been evaluated using these instruments.

To compare PM_2.5_ measurements for low exposures, or concentrations that may be representative of exposures in homes using intervention gas stoves, 39 field workers had three instruments (ECM, UPAS, and cyclone/pump) co‐located in the middle of their breathing zone (defined as on the chest halfway between the throat and diaphragm) while wearing a custom‐made kitchen apron with special pockets to hold the instruments together and keep the air inlets unobstructed and exposed (Figure 1D) to air. Field workers turned on instruments prior to entering study households and continued to have the machines run throughout study visits. Throughout the sampling period, field workers recorded the number of study households visited during daily follow‐ups, time spent in each household, and type of cookstove in the households. Low exposures were defined in our study as a mixture of short 5‐10‐minute visits spent in gas and/or traditional cookstove homes. Upon completion of study visits for the day, the field workers returned to the office and left the study apron hanging on coat hooks in the office overnight with all instrumentation inlets exposed until the machines turned off after 24 hours of sampling. All instruments (ECM, UPAS, and cyclone/pump) were run at 100% duty cycle, or continuous sampling.

To evaluate performance at medium levels of exposure (ie, HAP exposure levels representative of a typical biomass‐using primary cook in a Peruvian household), five study participants with daily biomass fuel use were enrolled and wore the same personalized study apron with the co‐located instruments that field workers used for low‐exposure assessment (Figure 1E). A total of 15 co‐location samples were collected. Consent forms were collected and signed at the time of enrollment, and participants were instructed to wear the study aprons for a 24‐hour time period, and to take off and place the aprons in the same room and breathing zone as themselves whether they were bathing or sleeping.^29^ The duty cycles on the ECM’s were changed to 30 seconds on, 30 seconds off (50% duty cycle) to avoid inlet blockages and battery life failures based on previous ECM testing results (unpublished data). The duty cycle on the UPAS and pump and cyclone remained at 100% (continuous sampling) due to the larger filter size used in both samplers (37 mm).

To evaluate performance at high levels of exposure (ie, exposures representative of large HAP concentrations from staying in a kitchen for an entire cooking event), birdcages containing an ECM, UPAS, and cyclone/pump were installed in a kitchen at one meter horizontally and 1.5 m vertically in height from the cookstove combustion zone.^29^ A total of 28 co‐location samples were collected. An example installation is shown in Figure 1F. We visited each of these households and collected their area concentration measurements three times over the study period for repeated measurements, since we assumed that day‐to‐day sampling variation was sufficient for independent samples.^20^ Due to the high pollution concentrations in the kitchens, the ECM duty cycle was changed to 20 seconds on, 160 seconds off (11.1% duty cycle) to avoid inlet blockages and battery life failures while the UPAS and cyclone/pump remained at 100% duty cycle as based on previous ECM testing results (unpublished data).

### 2.4 Sample and filter quality control

Flow rates for all instruments were measured and recorded before and after collecting each sample in the field. Field blanks, that is, filters placed into a sampler and installed in the household for 24 hours, while the sampler is off, were collected at a frequency of five times for every two weeks of sampling to account for background contamination that might occur during filter installation. Duplicate samples, that is, co‐located filters in two of the same type of instrument, were collected once out of every 10 area samples collected. Duplicates and field blanks were not able to be collected on study participants, as the additional weight of the apron would have likely affected participant compliance. Lastly, laboratory blanks, that is, filters that remain in the laboratory and are not deployed to the field, were also collected and sent with each batch of filters for analysis.

### 2.5 Filter assessment

Prior to being sent to Peru, filters were first conditioned in a climate‐controlled environment at the University of Georgia, Athens (UGA) (temperature range: 19.9‐20.1°C; relative humidity range: 39.7%‐42.5%) per Environmental Protection Agency laboratory guidelines.^31^, ^32^ In brief, the filters were placed in individual Petri dishes, laid out flat in the analysis clean room, and lids were cracked slightly ajar to allow slight airflow but not enough to allow particle/dust contamination to the filters. After conditioning in these settings for 48 hours, the filters were pre‐weighed prior to transport to Peru. After sampling in Puno, filters were stored in a freezer and then sent back to UGA for analysis using ice packs to keep the filters cool during transport. The filters were then equilibrated in the same controlled environment at UGA for 48 hours prior to post‐weighing. All filters were weighed twice on a scale with a sensitivity of one microgram with a weigh range of 250 mg and these weights were averaged (Cahn C‐35 Ultra‐Microbalance, Thermo Electron Corporation). The time‐weighted average of PM_2.5_ concentrations during sampling was calculated by dividing the total collected mass on the post‐weighed filter by the total volume of sampled air.

Samples were considered valid if the sampler ran for at least 23 hours and there was no visible damage to the collected filters. If flow rates after sampling differed by more than 15% from the calibrated flow rate (ECM = 0.3 LPM; UPAS = 1.0 liter per minute; Cyclone/Pump = 1.5 liter per minute), these samples were flagged. Limits of detection (LODs) were calculated for all three instruments types (ECM, UPAS, cyclone/pump) as three times the standard deviation of the mass weights for the 24 field blank filters collected: 8 filters per instrument with ECM, UPAS, and cyclone/pump LODs of 9, 28, and 14 µg, respectively.^33^ The gravimetric mass weights were then field blank weight‐corrected by subtracting the median field blank filter weight, as well as LOD‐corrected using the LOD mass weights described above. In brief, any filter mass measurements not exceeding the limit of detection were replaced by $\text{LOD}/\sqrt{2}$ in the calculation of the gravimetric mass concentration.^34^ If blank‐corrected, gravimetric filter weights fell below the LOD, they were excluded from the main analysis, but all LOD‐corrected values were used in the sensitivity analysis.

### 2.6 Biostatistical methods

The primary analytical objective was to assess agreement between PM_2.5_ concentrations measured by the ECM and the UPAS compared to the cyclone/pump method. This primary comparison analysis was done by using the Bland‐Altman method 35 to assess agreement and estimate bias in measurements. The Spearman correlation was calculated for both instruments to assess correlation^36^, ^37^ due to the non‐parametric nature of the collected data. These statistical analyses were first done using all data points, and then, further stratified by sampling environment, that is, high kitchen HAP concentrations versus low and medium personal HAP exposure measurements, to assess agreement and correlation. We performed these analyses with the assumption that all collocated instrument assessments were independent of each other and that the day‐to‐day sampling variability in PM_2.5_ measurements was enough to support this assumption.^20^ We conducted a sensitivity analysis where we included only co‐locations in which all three sampling instruments had no runtime or filter issues and in which all three instruments simultaneously collected samples with filters above the LOD to see how this selection would affect results. The statistical programs STATA Version 15 (StataCorp) and R (R Foundation, http://www-rproject.org) were used to conduct these analyses.

Finally, as mentioned above, one advantage of the ECM device over the UPAS is its ability to collect and display real‐time nephelometric data that can be then be corrected against gravimetric filter data.^27^ Using the real‐time data collected by the ECMs, we were able to plot the hourly distribution of concentrations over the 24‐hour day collection period during this pilot study against the gravimetrically corrected real‐time data in each of the three exposure settings.^38^

## 3 RESULTS

### 3.1 Sample characteristics

For this study, there were a total of 82 instrument co‐location 24‐hour samples: 28 area (high‐exposure) co‐location samples and 54 personal co‐location samples collected (39 low‐exposure fieldworker measurements, 15 medium‐exposure primary cook measurements). Next, all samples with runtime or filter issues were dropped (ECM: five samples dropped for instrument issues, 33 samples dropped below LOD; UPAS: six samples dropped for instrument issues, 22 samples dropped below LOD; Cyclone/Pump: six samples dropped for instrument issues, seven samples dropped below LOD). The median filter weights were then calculated for each instrument and subtracted from the post‐sampling mass weight, and the LOD was calculated for each instrument type. The resulting samples used in the final analysis were 16 ECM and cyclone/pump area co‐locations with 21 ECM and cyclone/pump personal sample co‐locations for a total of 37 ECM and cyclone/pump co‐locations with gravimetric samples above LOD. For the UPAS, there were 21 UPAS and cyclone/pump area co‐locations and 28 personal sample co‐locations for a total of 49 UPAS and air cyclone/pump co‐locations. The summarized sampling results are included in Table S1, and the reasons for filter flagging and dropped samples are included in Table S2. There were no differences in sampling times for all samples (*P* = .72), area samples (*P* = 1.00), or personal samples (*P* = .66).

### 3.2 Average concentrations

All descriptive statistics by instrument are described in Table S3. In brief, all three instruments collected similar concentration ranges and averages during the 24‐hour co‐location experiments. All three instruments were able to collect samples over a large range of concentrations but also could handle very high particulate concentrations that are typically observed in indoor biomass‐user kitchens in our study area, as well as detect low concentrations. For the field blanks collected for each instrument, the median mass loading of these blanks was 1.5 µg, −3.2 µg, and −4 µg for the ECM, UPAS, and cyclone/pump, respectively.

### 3.3 Agreement between devices

**Figure 2:**
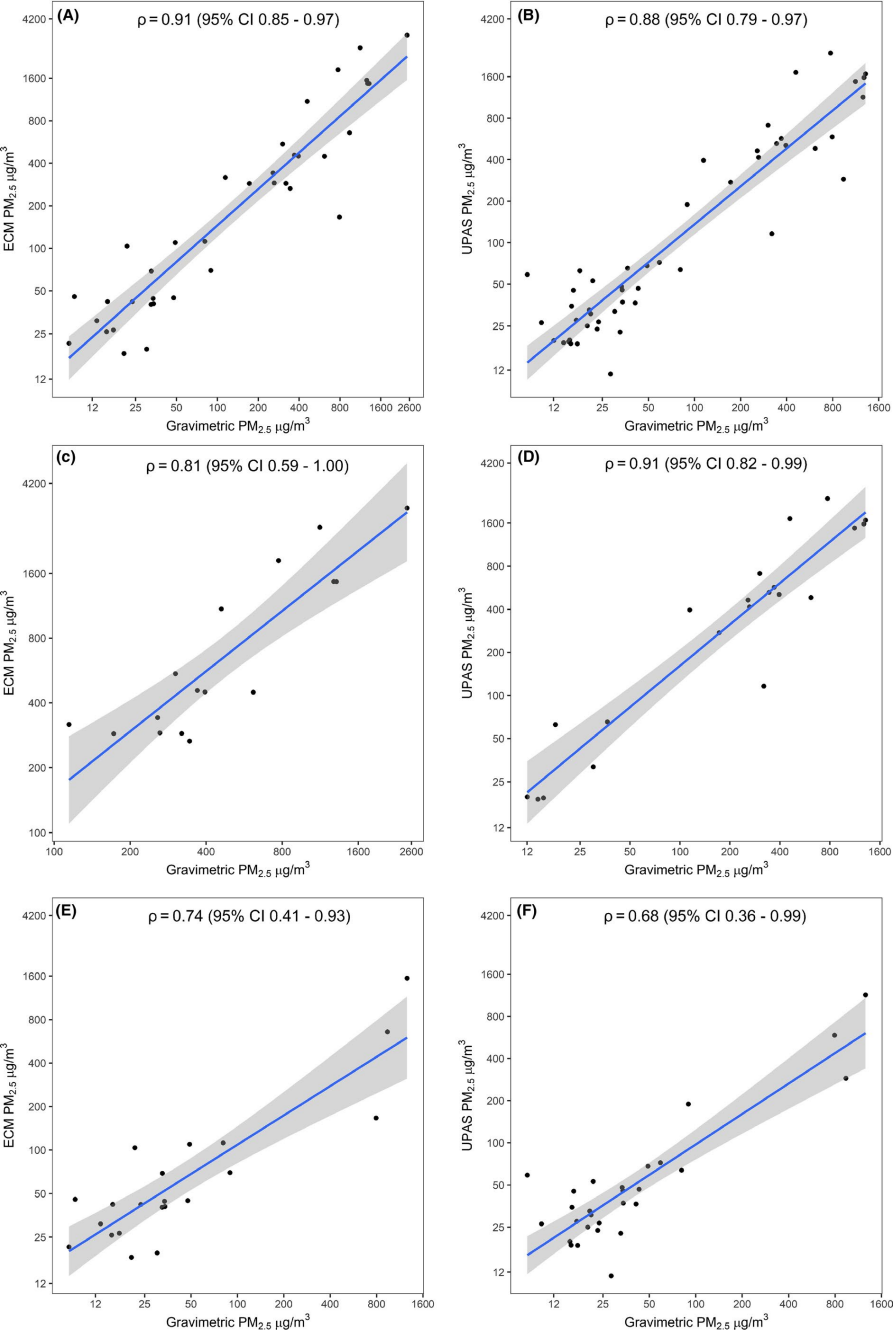
Correlation graphs comparing: A.) All samples (ECM vs cyclone and pump); B.) All samples (UPAS vs cyclone and pump); C.) Area samples (ECM vs cyclone and pump); D.) Area samples (UPAS vs cyclone and pump); E.) Personal samples (ECM vs pump and cyclone); F.) Personal samples (UPAS vs pump and cyclone)

**Figure 3:**
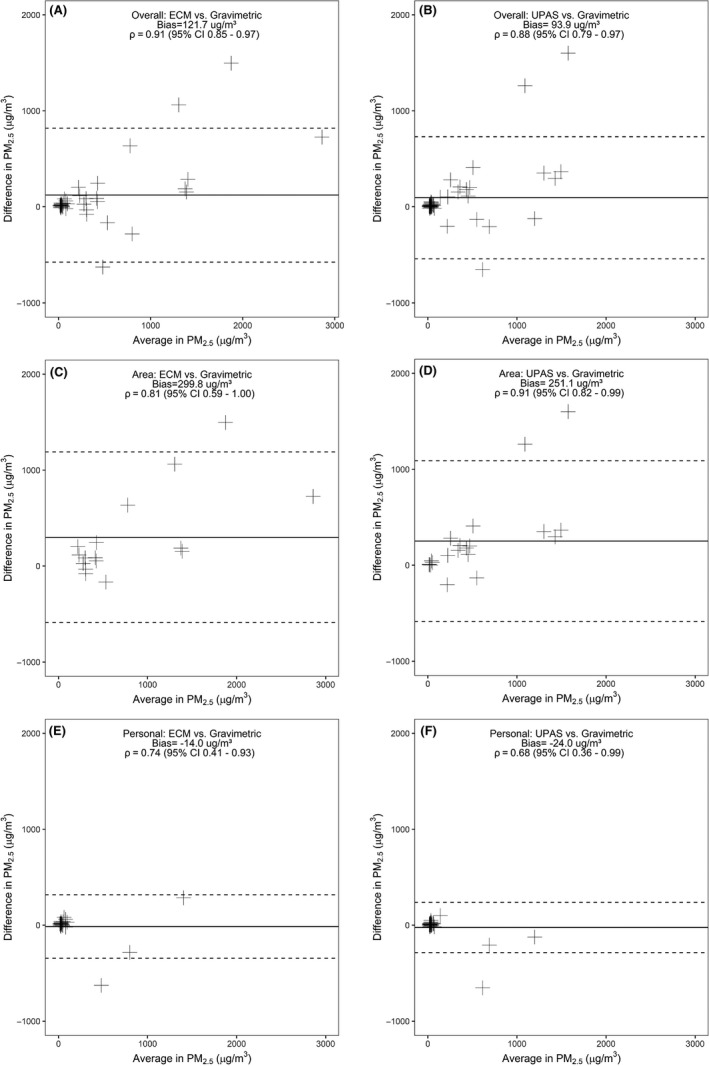
Bland‐Altman plots comparing: A.) All samples (ECM vs cyclone and pump); B.) All samples (UPAS vs cyclone and pump); C.) Area samples (ECM vs pump and cyclone); D.) Area samples (UPAS vs pump and cyclone); E.) Personal samples (ECM vs pump and cyclone); F.) Personal samples (UPAS vs pump and cyclone)

We present correlations and agreement between each next‐generation device and the cyclone/pump in Table S4, Figures 2, and 3. Overall, for both the area and personal samples, the ECM and the UPAS had similar agreement and correlation with overlapping confidence intervals for a majority of the Spearman's correlation coefficients and Bland‐Altman mean difference comparisons, when compared against the traditional gravimetric approach. The Spearman's correlation coefficient was slightly stronger overall for the ECM vs the cyclone/pump compared to that of the UPAS (ECM ρ = 0.91, 95% CI 0.85 to 0.97 vs UPAS ρ = 0.88, 95% CI 0.79‐0.97). However, the Bland‐Altman analysis showed overall better agreement between the UPAS and the cyclone/pump compared to the ECM (UPAS mean difference of 93.9 µg/m^3^ vs ECM mean difference of 121.7 µg/m^3^) (Figure 3A and 3B). Both instruments appear to have similar scattering patterns and numbers of outliers in the lower exposure ranges of the graphs, with the UPAS having only slightly tighter correlation in this range compared to the ECM (Figure 2A and 2B). Additionally, both instruments appeared to have tighter agreement in lower PM_2.5_ concentration ranges and more scattering in the higher concentration ranges (Figure 3A and 3B).

For area sampling, the UPAS correlation was better than the ECM correlation to the cyclone/pump (UPAS ρ = 0.91, 95% CI 0.82‐0.99 vs ECM ρ = 0.81, 95% CI 0.59‐1.00), but again with overlapping confidence intervals, the instruments appeared to perform similarly (Figure 2C and 2D). For the Bland‐Altman analysis, the UPAS once again had better agreement and less bias in measurements compared to the ECM (UPAS mean difference of 251.1 µg/m^3^ vs ECM mean difference of 299.8 µg/m^3^) (Figure 3C and 3D).

For personal sampling, the correlation between the ECM and the cyclone/pump was better compared to the UPAS (ECM ρ = 0.74, 95% CI 0.41‐0.93 vs UPAS ρ = 0.68, 95% CI 0.36‐0.99) (Figure 2E and 2F) and the agreement of the ECM vs cyclone/pump was also better compared to that of the UPAS vs cyclone/pump (ECM mean difference of −14.0 µg/m^3^ vs UPAS mean difference of −24.0 µg/m^3^) (Figure 3E and 3F). But again, with the overlapping confidence intervals for the Spearman correlation coefficients, similar Bland‐Altman mean differences (data not shown),^39^ and comparable scattering patterns and number of outliers in the Bland‐Altman plots (Table S4, Figure 2), these instruments performed similarly when used to measure personal exposure. In general, these results indicate that while the UPAS had lower bias overall across area samples and the ECM had lower bias in personal samples, both instruments performed comparably.^39^

In the first sensitivity analysis, a similar analysis was conducted to the original with the exclusion of outlier data points lying outside the original limits of agreement for the ECM vs cyclone/pump and the UPAS vs cyclone/pump measurements. In brief, we removed outliers that were outside the original overall Bland‐Altman limits of agreement for both instrument comparisons (ECM vs cyclone/pump limits of agreement −575.0 to 818.4 µg/m^3^, UPAS vs cyclone/pump limits of agreement −541.1 to 728.9 µg/m^3^). In detail, two area ECM outliers (ECM vs cyclone/pump differences of 1496.6 and 1062.3 µg/m^3^) and two area UPAS outliers (UPAS vs cyclone/pump differences of 1261.56 and 1601.2 µg/m^3^) were removed and the Spearman and Bland‐Altman analyses were re‐run.

Because the only samples that were dropped in this first sensitivity analysis were area samples, the personal correlation and agreement for the ECM vs cyclone/pump and UPAS vs cyclone/pump personal samples remained the same. Compared to the original analysis, the overall correlation between the ECM and the cyclone/pump remained relatively unchanged, but there was slightly lower correlation for area samples (overall ECM vs cyclone/pump samples ρ = 0.91, 95% CI 0.83‐0.98, area samples ρ = 0.75, 95% CI 0.40‐1.00). Overall correlation remained similar for the UPAS between this sensitivity analysis and the main analysis, but with a slightly stronger correlation for area samples (overall UPAS vs cyclone/pump samples ρ = 0.87, 95% CI 0.76‐0.98, area samples ρ = 0.92, 95% CI 0.79‐1.00).

In terms of bias, the ECM vs cyclone/pump magnitude of positive bias was more than halved compared to the main analysis (overall mean difference of 55.5 µg/m^3^, limits of agreement −372.3 to 483.4 µg/m^3^). Additionally, the positive bias was nearly halved for the area samples (area mean difference of 159.9 µg/m^3^, limits of agreement −325.7 to 645.5 µg/m^3^). Similarly, the overall UPAS vs cyclone/pump magnitude of positive bias was also more than halved compared to the main analysis (overall mean difference of 37.0 µg/m^3^, limits of agreement −286.1 to 360.2 µg/m^3^). This decrease was also observed for UPAS area samples (area mean difference of 126.9 µg/m^3^, limits of agreement −202.3 to 456.0 µg/m^3^). However, all the 95% confidence intervals overlapped for Spearman correlation statistics, and Bland‐Altman mean difference estimates were not significantly different from one another (data not shown),^39^ indicating that the instruments performed similarly to one another in sensitivity analysis. The results are detailed in Table S5.

Finally, in an additional sensitivity analysis in which all LOD‐corrected, imputed values that were not flagged were included, results slightly differed from the original main analysis. Compared to the original main analysis results, the overall correlation between the ECM and the cyclone/pump was lower, with a higher correlation for area samples, but the personal sample correlation was lower (overall ECM vs cyclone/pump samples ρ = 0.79, 95% CI 0.68‐0.91; area samples ρ = 0.93, 95% CI 0.78‐0.98; personal samples ρ = 0.56, 95% CI 0.32‐0.79). Correlation remained similar for the UPAS between the main and sensitivity analyses (overall UPAS vs cyclone/pump samples ρ = 0.86, 95% CI 0.76‐0.96; area samples ρ = 0.92, 95% CI 0.84‐1.00; personal samples ρ = 0.70, 95% CI 0.50‐0.91).

In terms of bias, the UPAS results did not appear to differ significantly between the main and this second sensitivity analysis but did decrease slightly for both area and personal samples (overall mean difference = 65.4 µg/m^3^, limits of agreement −467.1 to 597.9 µg/m^3^; area mean difference = 239.9 µg/m^3^, limits of agreement −583.1 to 1062.9 µg/m^3^; personal mean difference = −12.9 µg/m^3^, limits of agreement −211.8 to 186.0 µg/m^3^). For the ECM results, however, bias was generally lower compared to the main analysis when including these LOD‐corrected imputed values (overall mean difference = 68.8 µg/m^3^, limits of agreement −440.8 to 578.3 µg/m^3^). Similarly, bias was slightly lower in both area (mean difference = 223.4 µg/m^3^, limits of agreement −548.7 to 995.4 µg/m^3^) and personal samples (mean difference = −3.8 µg/m^3^, limits of agreement −218.7 to 211.0 µg/m^3^) for the ECM in this sensitivity analysis. However, even with these slight changes in Spearman's correlation values and magnitude of biases, all the 95% confidence intervals overlapped between the main and sensitivity analyses and the mean differences were not significantly different from one another (data not shown),^39^ indicating that there were no significant differences observed between the two analyses. We summarized these findings in Table S6.

### 3.4 Real‐time assessment

Using real‐time data collected by the ECMs, we plotted an hour‐by‐hour summary over the 24‐hour collection period against the gravimetrically corrected real‐time data in each of the three exposure settings. As an advantage of using the ECM over the UPAS, as displayed in Figure S1, we were able to visualize and summarize temporal cooking patterns of our participants, and peak exposure times and concentrations throughout the day. This figure shows peak concentrations of exceeding 500 µg/m^3^ during cooking hours, at around 6 am and 6 pm.

## 4 DISCUSSION

We collected 24‐hour gravimetric samples of an ECM and UPAS co‐located with a traditional cyclone/pump to measure air concentrations and personal exposures. Overall, we found that in our study setting of Peru, both the ECM and UPAS performed well and measured comparable concentrations to those from the traditional gravimetric instruments. The overall bias in either device undergoing testing was estimated to be approximately less than 10% of the measurement concentration range obtained from the traditional gravimetric device (ECM overall bias: 121.7 µg/m^3^, UPAS overall bias: 93.9 µg/m^3^ out of 1303.2 µg/m^3^ range of concentration measurements for the pump and cyclone). This bias is within range for these types of air sampling instruments, as calibration comparisons of the traditional pump and cyclone used in this study compared to reference standards sampling at similar flow rates and cut‐points have been shown to have a bias of 10% between themselves. Within the two sets of duplicate cyclone/pump samples collected for this study, the percent differences in concentrations collected were approximately two percent and seven percent (data not shown).

In this study, we performed a co‐location comparison of the ECM and UPAS against a traditional gravimetric instrument commonly used in field studies. However, past studies have sought to evaluate both laboratory and field performance of just one of these instruments or past versions of the instruments compared to commonly used gravimetric instruments.^18^, ^25^, ^40^, ^41^, ^42^, ^43^, ^44^, ^45^, ^46^, ^47^, ^48^ Although there are no evaluation studies to date that have been conducted on the current version of the ECM, except for a pilot study to compare the ECM to the MicroPEM,^40^ several studies have been done with an older version of RTI’s MicroPEM. There are several field studies that have used the MicroPEM to assess associations of PM_10_ to mold exposure in asthmatic children in Denver, CO,^42^ assess whether ambient sampling instruments approximate personal exposures^45^ and compare outdoor PM to indoor PM exposures in children living in Utah,^43^ and measure emissions from wood stoves in Norway.^44^ However, there are only a few studies that have evaluated the MicroPEM’s performance against traditional gravimetric field instruments. Guak and Lee investigated the correlation between personal and ambient concentrations of PM_10_ and PM_2.5_ by co‐locating RTI’s MicroPEM (v3.2) against central ambient air monitors for PM_10_ and PM_2.5_. The MicroPEM showed a strong linear relationship with the co‐located monitors (PM_10_ *R* ^2^ = 0.89, PM_2.5_ *R* ^2^ = 0.93), similar to the ECM vs cyclone/pump results we obtained in our study.^46^ The US Environmental Protection Agency (EPA) also conducted a series of experiments to evaluate the MicroPEM versus the Grimm Model EDM180 PM_2.5_ FEM air monitor. The MicroPEM had variable correlation with the Grimm ranging from *R* ^2^ = 0.54‐0.88, with an average experimental *R* ^2^ = 0.72.^47^ These correlations were similar to the values we obtained.

The only published cookstove field study that used the MicroPEM was conducted in Kopiwatta, Sri Lanka and collected 48‐hour, real‐time indoor concentrations and personal exposures of the primary cook to PM_2.5_ in 53 households.^41^ The authors compared exposures between households with traditional or improved (Anagi) stoves with chimneys present or absent. The concentrations measured for indoor PM_2.5_ ranged from 33 to 940 µg/m^3^ and personal exposures ranged from 34 to 522 µg/m^3^.^41^ While these concentrations were lower than the measurements we collected for our study in Peru using the ECM (indoor area concentration range = 265.3‐3222.7 µg/m^3^, personal exposure concentration range = 18.1‐1546.7 µg/m^3^), a few reasons for these observed values could potentially be attributed to the lower oxygen concentration at higher altitude^49^ leading to less efficient fuel combustion,^50^ higher amount of fuel use needed for heating the home due to the colder Puno climate, or use of different biomass materials with different characteristics (ie, moisture content) for cooking fuel (firewood in Sri Lanka vs crop waste/animal dung in Puno). Overall, more co‐location field studies with gravimetric standard instrumentation need to be done to properly evaluate the newer ECM model for future use.

In comparison to the ECM, there have been more gravimetric and performance evaluation studies conducted with the UPAS. Volkens et al (2016) conducted a laboratory evaluation of the first UPAS prototype against a federal reference method (FRM) for PM_2.5_ monitoring and a personal environmental monitor (PEM) to measure PM_2.5_ personal exposure. After co‐locating all three instruments in three locations of an aerosol test chamber (nine samplers in total) and exposing all of them to standard generated aerosols, the UPAS showed strong agreement with the EPA FRM. The linear regression slope between the UPAS and the FRM resulted in 0.986 with an intercept of 3.7 µg/m^3^, which was a comparable result with the regression of the PEM sampler against the FRM (slope = 0.959, intercept = 11.5 µg/m^3^). The average difference between the UPAS and the FRM was 7% compared to 6% for the PEM and FRM with no directional bias observed.^25^ For a field evaluation study, Pillarisetti et al’s (2018) results indicated that the UPAS could be a viable option for assessing personal exposure to PM_2.5_ household air pollution. After collecting 43 co‐location samples of a UPAS and a personal exposure sampling setup similar to the one used in our study (SCC 1.062 Triplex Cyclone with AirChek XR5000), the correlation and agreement between the two instruments were very high (Spearman ρ = 0.91 and bias of 7.7 µg/m^3^).^18^ Finally, another recent field evaluation conducted by Arku et al to inform the Prospective Urban Rural Epidemiology (PURE) study collected 43 kitchen co‐located samples of a UPAS and a reference Harvard Impactor (Boston, MA) in rural India. Correlation between the two instruments was also found to be strong in this preliminary field study (Pearson's *r* = 0.91, 95% CI 0.84‐0.95).^48^ These correlation results are similar to the ones obtained in our study in which we observed an overall Spearman's correlation coefficient of ρ = 0.88 and a personal sample correlation coefficient of ρ = 0.68, but we did observe a larger bias for overall sampling (mean difference = 93.9 µg/m^3^) and slightly larger negative bias for personal samples (mean difference = −24.0 µg/m^3^). The potential explanations for these differences may be due to the same reasons described for the ECM measurements above, but future evaluation studies for the UPAS are also needed.

Our study had several strengths. We evaluated two new air quality instruments, the ECM and the UPAS, against a traditional gravimetric field instrument over a large range of personal exposure and area concentrations. As cited above, past studies have typically only evaluated one of these instruments against gravimetric standards and in laboratory settings. By conducting this study in the field, this gives us a better understanding of real‐life performance^21^, ^22^ in low‐resource settings with non‐technical field staff, potential issues involved with field transportation of the instruments to the laboratory, and filter processing and handling problems once the filters are transported back to the United States. Another valuable piece of information gained from this study was that there was an observed difference in measurement error, or mean difference, between area concentration and personal exposure measurements that can be potentially corrected for in future dose‐response analyses after data collection. Further, the correlations with personal samples, most important for detecting valid associations with health outcomes were decent and the mean differences were small, thus holding promising potential for future exposure‐response analyses. Lastly, another strength of this study included comparing two instruments instead of one against a traditional gravimetric sampling instrument, thus allowing us to objectively compare them and modify our current HAPIN trial protocols to collect high‐quality measurements for personal exposure or area monitoring.

Potential shortcomings included a relatively small sample size (n = 82 samples) and a small number of area samples (n = 23) with co‐locations conducted in only six households with repeated measures. We recognize that the number of samples collected is a limitation and that a larger sample size would help reduce the likelihood of repeated measures influencing the area concentration measurement results. However, we believe that the day‐to‐day variability in area measurements that have been observed in past studies^20^ is high enough, thus decreasing the possibility that the measurements are not independent. Second, we believe that while the ECM and UPAS had higher observed concentrations compared to those seen in the cyclone/pump samples, these results are driven by the area high‐exposure samples and, in that sense, may not be relevant to our original goal of obtaining better data on low‐exposure personal exposures. A possible explanation as to why the Spearman correlation and Bland‐Altman results for the area samples are so discrepant between the main analysis and the first sensitivity analysis may be due to the small number of area samples collected and our Bland‐Altman mean difference results were likely driven by a small number of outliers in these plots. As shown by the results in our first sensitivity analysis that did not include two outstanding ECM and two outstanding UPAS samples, while the correlation decreased for the ECM vs cyclone/pump measurements, the Bland‐Altman mean difference significantly decreased in magnitude overall and for the area samples for both the ECM and the UPAS. In addition to the duty cycle difference between sample types, any single monitor may have been influenced by placement in the kitchen (ie, one inlet is more exposed than others, even though they are close together), as well as instrument operation‐specific issues. Third, personal exposures (both “low” and “medium” as designated in our manuscript) are typically lower, sometimes much lower, than area concentrations, thus leading to a greater number of samples being represented in these lower concentration ranges compared to higher area concentrations. Finally, this analysis was only conducted using data from Peru and thus more testing needs to be conducted in other LMIC’s.

In terms of the differences in results observed between the main and sensitivity analyses, potential explanations for these changes in correlation and bias are four‐fold. During this period of testing, based on what we had observed for biomass stove area monitoring and ECM filter clogging issues in our previous CHAP study,^29^ we instituted 50% (primary cook monitor) and 11.11% (area monitor) duty cycling in the ECM’s that were installed in biomass‐using homes. However, because this study only conducted 24‐hour sampling compared to CHAP’s 48‐hour sampling regimen, this shortened amount of sampling time may explain why so many of the ECM’s filters fell below the LOD cutoff (n = 33, 45%) compared to the UPAS (n = 22, 31%) and the cyclone/pump (n = 7, 10%), as well as the variability in the household sampling time necessary to obtain the LOD saturation exposure. Second, due to the ECM’s filter size (15 mm) in combination with the duty cycling, compared to the larger UPAS filter (37 mm) with 100% duty cycling for all samples, the differential testing regimen between the two instruments for only 24 hours of sampling may have led to insufficient mass loading for the ECM. Third, it is unknown whether the 15 mm filter size may have clogged sooner than the 37 mm filters used in the cyclone/pump setup and the UPAS due to smaller surface area in highly polluted kitchen environments. However, since the amount of bias was similar between the ECM and the UPAS, the filter size may not have been a major issue overall. Lastly, in our particular field setting, we saw that the staff had had more experience using the ECM’s compared to the UPAS due to their familiarity with using them in our CHAP study.^29^ Due to the novelty and unfamiliarity of using the UPAS in our field setting, more attention may have been paid to the filter handling and processing steps of cleaning the UPAS compared to the ECM, as observed by the number of UPAS samples above LOD.

## 5 CONCLUSIONS

The ECM and the UPAS both performed well overall and produced comparable agreement and mean difference statistics with overlapping confidence intervals to the same traditional gravimetric sampling instrument. Due to the sample size collected in our study, more data would be helpful to determine the performance of these instruments at lower concentrations.

## Appendix 1

### Household Air Pollution Intervention Network (HAPIN) Investigators (listed in alphabetical order)

- HAPIN Steering Committee: Kalpana Balakrishnan (Sri Ramachandra Institute for Higher Education and Research, Chennai, India), William Checkley (Johns Hopkins University, Baltimore, MD, USA), Thomas Clasen (Emory University, Atlanta, GA, USA), John McCracken (Universidad del Valle de Guatemala, Guatemala City, Guatemala), Jennifer Peel (Colorado State University, Ft. Collins, CO, USA), Ghislaine Rosa (London School of Hygiene and Tropical Medicine, London, UK), Joshua Rosenthal (Fogarty International Center, National Institutes of Health, Bethesda, MD, USA), Kyle Steenland (Emory University, Atlanta, GA, USA), Lisa Thompson (Emory University, Atlanta, GA, USA).
- HAPIN India research center: Vigneswari Aravindalochanan (Sri Ramachandra Institute for Higher Education and Research, Chennai, India), Kalpana Balakrishnan (Sri Ramachandra Institute for Higher Education and Research, Chennai, India), Sarada Garg (Sri Ramachandra Institute for Higher Education and Research, Chennai, India), Gurusamy Thangavel (Sri Ramachandra Institute for Higher Education and Research, Chennai, India), Krishnendu Mukhopadhyay (Sri Ramachandra Institute for Higher Education and Research, Chennai, India), Naveen Puttaswamy (Sri Ramachandra Institute for Higher Education and Research, Chennai, India), Sankar Sambandam (Sri Ramachandra Institute for Higher Education and Research, Chennai, India),
- HAPIN Guatemala research center: Oscar De Leon (Universidad del Valle de Guatemala, Guatemala City, Guatemala), Erick Mollinedo (Universidad del Valle de Guatemala, Guatemala City, Guatemala), Anaité Diaz (Universidad del Valle de Guatemala, Guatemala City, Guatemala), Irma Sayury Pineda Fuentes (Universidad del Valle de Guatemala, Guatemala City, Guatemala), John McCracken (Universidad del Valle de Guatemala, Guatemala City, Guatemala), Lisa Thompson (Emory University, Atlanta, GA, USA).
- HAPIN Peru research center: Vanessa Burrowes (Johns Hopkins University, Baltimore, MD, USA), Eduardo Canuz (Asociación Benéfica PRISMA, Lima, Peru), William Checkley (Johns Hopkins University, Baltimore, MD, USA), Marilú Chiang (Asociación Benéfica PRISMA, Lima, Peru), Juan Gabriel Espinoza Asociación Benéfica PRISMA, Lima, Peru), Stella Hartinger (Universidad Peruana Cayetano Heredia, Lima, Peru), Phabiola Herrera (Johns Hopkins University, Baltimore, MD, USA), Margaret Laws (Johns Hopkins University, Baltimore, MD, USA), Lawrence Moulton (Johns Hopkins University, Baltimore, MD, USA), Suzanne Simkovich (Johns Hopkins University, Baltimore, MD, USA), Lindsay Underhill (Johns Hopkins University, Baltimore, MD, USA), Kendra Williams (Johns Hopkins University, Baltimore, MD, USA).
- HAPIN Rwanda research center: Jean de Dieu Ntivuguruzwa (Eagle Research Center, Kayonza, Rwanda), Miles Kirby (Emory University, Atlanta, GA, USA), Fiona Majorin (London School of Hygiene and Tropical Medicine, London, UK), Abidan Nambajimana (Eagle Research Center, Kayonza, Rwanda), Florien Ndagijimana (Eagle Research Center, Kayonza, Rwanda), Ghislaine Rosa (London School of Hygiene and Tropical Medicine, London, UK), Jean Uwizeyimana (Eagle Research Center, Kayonza, Rwanda).
- HAPIN Behavioral Economics Core: Steven Harvey (Johns Hopkins University, Baltimore, MD, USA), Marjorie Howard (Emory University, Atlanta, GA, USA), J. Jaime Miranda (Universidad Peruana Cayetano Heredia, Lima, Peru), Elisa Puzzolo (Global LPG Partnership, New York, NY, USA), Ashlinn Quinn (Fogarty International Center, National Institutes of Health, Baltimore, MD, USA), Zoe Sakas (London School of Hygiene and Tropical Medicine, London, UK), Kendra Williams (Johns Hopkins University, Baltimore, MD, USA)
- HAPIN Biomarker Core: Dana Barr (Emory University, Atlanta, GA, USA), Julia McPeek Campbell (Emory University, Atlanta, GA, USA), Maggie Clark (Colorado State University, Ft. Collins, CO, USA), Savannah Gupton (Emory University, Atlanta, GA USA), Sarah Rajkumar (Colorado State University, Ft. Collins, CO, USA), Barry Ryan (Emory University, Atlanta, GA, USA), Bonnie Young (Colorado State University, Ft. Collins, CO, USA).
- HAPIN Data Management Core: Howard Chang (Emory University, Atlanta, GA, USA), Yunyun Chen (Emory University, Atlanta, GA, USA), Lisa Elon (Emory University, Atlanta, GA, USA), Lindsay Jaacks (Harvard University, Cambridge, MA, USA), Shirin Jabbarzadeh (Emory University, Atlanta, GA, USA), Amy Lovvorn (Emory University, Atlanta, GA, USA), Azhar Nizam (Emory University, Atlanta, GA, USA), Amit Verma (Emory University, Atlanta, GA, USA), Lance Waller (Emory University, Atlanta, GA, USA).
- HAPIN Clinical and Imaging Core: William Checkley (Johns Hopkins University, Baltimore, MD, USA), Rachel Craik (University of Oxford, Oxford, UK), Rachel Merrick (Washington University in St. Louis, St. Louis, MO, USA), Victor Davila‐Roman (Washington University in St. Louis, St. Louis, MO, USA), Lisa de la Fuentes (Washington University in St. Louis, St. Louis, MO, USA), Aris Papageorghiou (University of Oxford, Oxford, UK), Ashley Toenjes (Washington University in St. Louis, St. Louis, MO, USA)
- HAPIN Exposure Core: Michael Johnson (Berkeley Air Monitoring Group, Berkeley, CA, USA), Jiawen Liao (Emory University, Atlanta, GA, USA), Luke Naeher (University of Georgia, Athens, GA, USA), Ricardo Piedrahita (Berkeley Air Monitoring Group, Berkeley, CA, USA), Ajay Pillarisetti (University of California, Berkeley, CA, USA), Jeremy Sarnat (Emory University, Atlanta, GA, USA), Kirk Smith (University of California, Berkeley, Berkeley, CA, USA),
- HAPIN Nutrition Working Group: Marilú Chiang (Asociación Benéfica PRISMA, Lima, Peru), Anaité Diaz (Universidad del Valle de Guatemala, Guatemala City, Guatemala), Lindsay Jaacks (Harvard University, Cambridge, MA, USA), Miles Kirby (Emory University, Atlanta, GA, USA), Usha Ramakrishnan (Emory University, Atlanta, GA, USA), Lisa Thompson (Emory University, Atlanta, GA, USA), Sheela Sinharoy (Emory University, Atlanta, GA, USA).
- HAPIN Pneumonia Working Group: William Checkley (Johns Hopkins University, Baltimore, MD, USA), Thomas Clasen (Emory University, Atlanta, GA, USA), Mary Crocker (University of Washington, Seattle, WA, USA), Dina Goodman (Johns Hopkins University, Baltimore, MD, USA), Shakir Hossen (Johns Hopkins University, Baltimore, MD, USA), Miles Kirby (Emory University, Atlanta, GA, USA), Eric McCollum (Johns Hopkins University, Baltimore, MD, USA), John McCracken (Universidad del Valle de Guatemala, Guatemala City, Guatemala), Jennifer Peel (Colorado State University, Ft. Collins, CO, USA), Ghislaine Rosa (London School of Hygiene and Tropical Medicine, London, UK), Suzanne Simkovich (Johns Hopkins University, Baltimore, MD, USA), Kyle Steenland (Emory University, Atlanta, GA, USA), Sarada Garg (Sri Ramachandra Institute for Higher Education and Research, Chennai, India), Gurusamy Thangavel (Sri Ramachandra Institute for Higher Education and Research, Chennai, India), Lisa Thompson (Emory University, Atlanta, GA, USA).
- HAPIN Project Management: Eduardo Canuz (Asociación Benéfica PRISMA, Lima, Peru), Anaité Diaz (Universidad del Valle de Guatemala, Guatemala City, Guatemala), Margaret Laws (Johns Hopkins University, Baltimore, MD, USA), Amy Lovvorn (Emory University, Atlanta, GA, USA), Phabiola Herrera (Johns Hopkins University, Baltimore, MD, USA), Gurusamy Thangavel (Sri Ramachandra Institute for Higher Education and Research, Chennai, India), Jean Uwizeyimana (Eagle Research Center, Kayonza, Rwanda).
