# Supplementary figures and images for: Comparison of next‐generation portable pollution monitors to measure exposure to PM2.5 from household air pollution in Puno, Peru
Source: Indoor Air. 2020 Jan 23;30(3):445–58. doi: 10.1111/ina.12638 (PMC7217081; doi:10.1111/ina.12638)

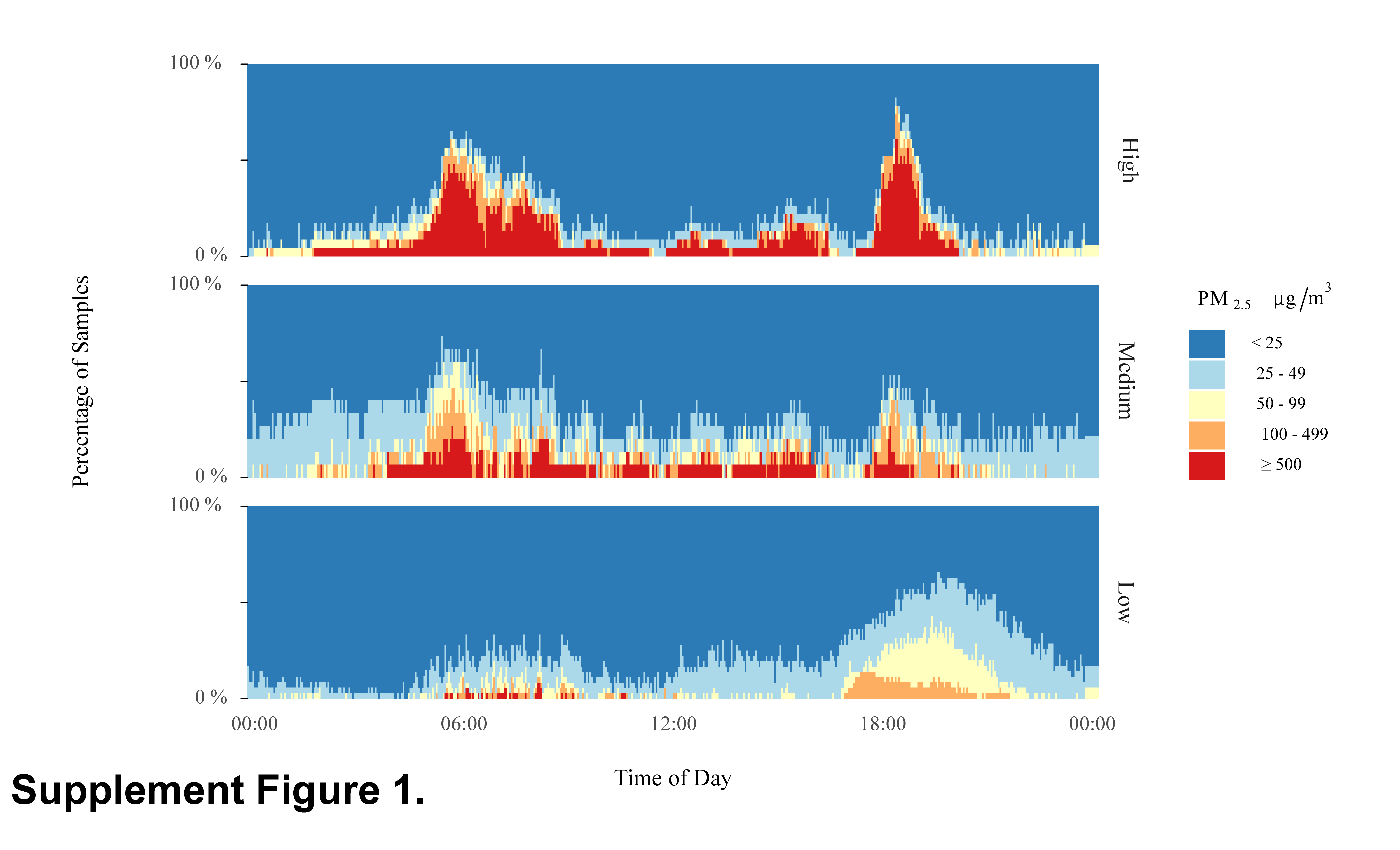

Supplement: Supplementary file 1 [file INA-30-445-s001.tiff]
