# Supplementary material for: Comparison of next‐generation portable pollution monitors to measure exposure to PM2.5 from household air pollution in Puno, Peru
Source: Indoor Air. 2020 Jan 23;30(3):445–58. doi: 10.1111/ina.12638 (PMC7217081; doi:10.1111/ina.12638)
Supplement: Supplementary file 2 [file INA-30-445-s002.docx]

**APPENDIX**

**Supplement Table 1. Summary description of exposure assessment sampling schematic (using all collected samples above LOD)**

|  | Number of ECM/Gravimetric Co-locations | Number of UPAS/Gravimetric Co-locations | Total Number of Surveyed People/Kitchens | Duty Cycle (%) of ECM | Duty Cycle (%) of UPAS | Duty Cycle (%) of Cyclone |
| --- | --- | --- | --- | --- | --- | --- |
| Low-Exposure Measurements | 12 | 17 | 33 | 100 | 100 | 100 |
| Medium Exposure Measurements | 9 | 11 | 5 | 50 | 100 | 100 |
| High Exposure Measurements | 16 | 21 | 6 | 11.11 | 100 | 100 |

**Supplement Table 2. Summary of instrument run-time and filter flagging issues for ECM, UPAS, and pump/cyclone set-ups**

| **ECM Sample Flagging Issues (n=5 samples)** |
| --- |
| 1.) Small hole found in filter |
| 2.) ECM only found to have been running for 12 hours |
| 3.) ECM machine showed “Errored Use” message and shut down for 3 minutes during sampling, likely due to pump failure |
| 4.) ECM found to not be functioning at 10 AM on 9/20/17 after having started sampling 5 hours prior and had to be turned off manually at 5:10 AM 9/21/17 |
| 5.) ECM found running in a Ziploc bag since 1 AM, needed to redo the sample |
| **UPAS Sample Flagging Issues (n=6 samples)** |
| 1.) UPAS machine only ran until 1:45 AM |
| 2.) Machine was found turned off when picked up at 4:05 AM |
| 3.) Small hole in post-sampling filter |
| 4.) UPAS turned off for 1 hour during sampling |
| 5.) Hole found near edge of post-sampled filter |
| 6.) UPAS found to not be running when found at 3:40 PM and had to be restarted and turned off manually at 5:10 AM |
| **Pump and Cyclone Flagging Issues (n=6 samples)** |
| 1.) 144 minutes were found to be remaining on the machine at pickup |
| 2.) Cyclone was found at 6:50 AM, had turned itself off with 143 mins remaining, fieldworker turned instrument on again until all other instruments stopped, and the final sample had 86 mins remaining. The filter was also flagged for having a large hole |
| 3.) Post-weighed filter has a large hole |
| 4.) Cyclone was found to be turned off at 6:40 and fieldworker had to return to restart it |
| 5.) Post-weighed filter flagged for having a hole near the edge of the filter |
| 6.) Post-sampling filter had small hole near the edge of the filter |

**Supplement Table 3. Descriptive characteristics summary of personal and area study measurements above LOD**

|  | ECM (n=37) | UPAS (n=49) | Gravimetric Pump and Cyclone (n=49) |
| --- | --- | --- | --- |
| Instrument Limit of Detection Mass Weight (µg) | 9 | 28 | 14 |
| Overall IQR (μg/m^3^) | 414.4 | 436.3 | 300.1 |
| Overall Conc. Range Collected for All Samples (μg/m^3^) | 18.1 – 3222.7 | 11.3– 2375.3 | 8.1 – 1311.3 |
| Average, Median Conc. Collected for All Samples (μg/m^3^) | 504.7, 166.4 | 338.7, 58.7 | 244.8, 36.6 |
| IQR of Area Samples (μg/m^3^) | 1155.0  (n=16) | 646.2  (n=21) | 422.8  (n=21) |
| Overall Conc. Range Collected for Area (μg/m^3^) | 265.3 – 3222.7 | 18.9 – 2375.3 | 12.1 – 1311.3 |
| Average, Median Area Concentration (μg/m^3^) | 962.5, 452.8 | 643.1, 463.8 | 391.9, 302.5 |
| IQR of Personal Samples (μg/m^3^) | 72.7  (n=21) | 33.9  (n=28) | 27.3  (n=28) |
| Overall Exposure Range Collected for Personal Samples (μg/m^3^) | 18.1 – 1546.7 | 11.27 – 1137.70 | 8.1 – 1261.6 |
| Average, Median Personal Exposure (μg/m^3^) | 156.0, 44.2 | 110.51, 36.82 | 134.5, 26.0 |

**Supplement Table 4: Summary of Spearman’s and Bland-Altman statistical analysis findings for all samples above LOD**

|  | ECM - Gravimetric (n=37) | UPAS - Gravimetric (n=49) |
| --- | --- | --- |
| Overall Spearman Rho (All Area and Personal Samples) (95% CI) | ρ = 0.91  (0.85 – 0.97) | ρ = 0.88  (0.79 – 0.97) |
| Overall Bland-Altman Limits of Agreement (95% CI)  (All Area and Personal Samples) | 121.7 µg/m^3^ (-575.0 µg/m^3^ – 818.4 µg/m^3^) | 93.9 µg/m^3^ (-541.1 µg/m^3^ – 728.9 µg/m^3^) |
| Area Samples Spearman Rho  (95% CI) | ρ = 0.81  (0.59 – 1.00) | ρ = 0.91  (0.82 – 0.99) |
| Area Samples Bland-Altman Limits of Agreement (95% CI) | 299.8 µg/m^3^ (-589.2 µg/m^3^ – 1188.8 µg/m^3^) | 251.1 µg/m^3^  (-585.6 µg/m^3^ – 1087.8 µg/m^3^) |
| Personal Samples Spearman Rho (95% CI) | ρ = 0.74  (0.41 – 0.93) | ρ = 0.68  (0.36 – 0.99) |
| Personal Samples Bland-Altman Limits of Agreement  (95% CI) | -14.0 µg/m^3^  (-344.3 µg/m^3^ – 316.3 µg/m^3^) | -24.0 µg/m^3^  (-286.7 µg/m^3^ – 238.8 µg/m^3^) |

**Supplement Table 5. Secondary Sensitivity Analysis – Summary of Spearman correlation and Bland-Altman statistical analysis findings for all samples above LOD minus outlier area samples**

|  | ECM - Gravimetric (n=35) | UPAS - Gravimetric (n=47) |
| --- | --- | --- |
| Overall Spearman Rho (All Area and Personal Samples) (95% CI) | ρ = 0.91  (0.83 – 0.98) | ρ = 0.87  (0.76 – 0.98) |
| Overall Bland-Altman  Limits of Agreement (95% CI)  (All Area and Personal Samples) | 55.5 µg/m^3^ (-372.3 µg/m^3^ – 483.4 µg/m^3^) | 37.0 µg/m^3^ (-286.1 µg/m^3^ – 360.2 µg/m^3^) |
| Area Samples Spearman Rho  (95% CI) | ρ = 0.75 (0.40 – 1.00) | ρ = 0.92  (0.79 – 1.00) |
| Area Samples Bland-Altman Limits of Agreement (95% CI) | 159.9 µg/m^3^ (-325.7 µg/m^3^ – 645.5 µg/m^3^) | 126.9 µg/m^3^  (-202.3 µg/m^3^ – 456.0 µg/m^3^) |
| Personal Samples Spearman Rho (95% CI) | ρ = 0.74  (0.41 – 0.93) | ρ = 0.68  (0.36 – 0.99) |
| Personal Samples Bland-Altman Limits of Agreement  (95% CI) | -14.0 µg/m^3^  (-344.3 µg/m^3^ – 316.3 µg/m^3^) | -24.0 µg/m^3^  (-286.7 µg/m^3^ – 238.8 µg/m^3^) |

**Supplement Table 6 Sensitivity Analysis – Summary of Spearman’s Correlation and Bland-Altman statistical analysis findings: All samples with LOD-corrections that had no sampling issues.**

|  | ECM - Gravimetric (n=72) | UPAS - Gravimetric (n=71) |
| --- | --- | --- |
| Overall Spearman Rho (All Area and Personal Samples) (95% CI) | ρ = 0.79  (0.68 – 0.91) | ρ = 0.86  (0.76 – 0.96) |
| Overall Bland-Altman Limits of Agreement (95% CI)  (All Area and Personal Samples) | 68.8 µg/m^3^ (-440.8 µg/m^3^ – 578.3 µg/m^3^) | 65.4 µg/m^3^ (-467.1 µg/m^3^ – 597.9 µg/m^3^) |
| Area Samples Spearman Rho  (95% CI) | ρ = 0.93 (0.78 – 0.98) | ρ = 0.92  (0.84 – 1.00) |
| Area Samples Bland-Altman Limits of Agreements (95% CI) | 223.4 µg/m^3^ (-548.7 µg/m^3^ – 995.4 µg/m^3^) | 239.9 µg/m^3^  (-583.1 µg/m^3^ – 1062.9 µg/m^3^) |
| Personal Samples Spearman Rho (95% CI) | ρ = 0.56  (0.32– 0.79) | ρ = 0.70  (0.50 – 0.91) |
| Personal Samples Bland-Altman Limits of Agreement  (95% CI) | -3.8 µg/m^3^  (-218.7 µg/m^3^ – 211.0 µg/m^3^) | -12.9 µg/m^3^  (-211.8 µg/m^3^ – 186.0 µg/m^3^) |
